# Expression of NF-κB and IL-6 in oral precancerous and cancerous lesions: An immunohistochemical study

**DOI:** 10.4317/medoral.20570

**Published:** 2015-11-22

**Authors:** Georgios Kamperos, Nikolaos Nikitakis, Aikaterini Sfakianou, Dimitrios Avgoustidis, Alexandra Sklavounou-Andrikopoulou

**Affiliations:** 1 DDS, MSc. Postgraduate student, Department of Oral Medicine and Pathology, School of Dentistry, University of Athens, Greece; 2 MD, DDS, PhD. Associate Professor, Department of Oral Medicine and Pathology, School of Dentistry, University of Athens, Greece; 3 MD, DDS. Resident, Department of Oral and Maxillofacial Surgery, “Evaggelismos” Hospital, University of Athens, Greece; 4 DDS, MSc, DrDent. Professor and Head, Department of Oral Medicine and Pathology, School of Dentistry, University of Athens, Greece

## Abstract

**Background:**

The purpose of this study was to evaluate the immunohistochemical expression of NF-κB and IL-6 in oral premalignant and malignant lesions and to investigate their possible correlation with the presence of subepithelial inflammation.

**Material and Methods:**

Thirty two oral premalignant lesions, clinically compatible with leukoplakia or erythroplakia, were investigated. Microscopically, 11 of them showed hyperkeratosis and acanthosis (epithelial hyperplasia) and 21 showed dysplasia of varying degrees. Nine cases of OSCC and four control cases of normal oral mucosa were also included in the study. Immunohistochemical staining with NF-κB (p65) and IL-6 was performed. IL-6 and nuclear NF-κB staining were assessed as positive or negative. For cytoplasmic localization of NF-κB, a total score combining intensity and percentage of positive epithelial cells was additionally calculated. The presence of inflammation was also recorded.

**Results:**

Intensity and total scores for NF-κΒ cytoplasmic immunostaining showed a statistically significant gradual increase from normal mucosa to OSCC (*p*=0.012 and *p*=0.026 respectively). Non-statistically significant increased NF-κΒ nuclear localization was detected in dysplasias and OSCCs. Positive statistical correlation was detected between the presence of inflammation and IL-6 expression (*p*=0.015). No correlation between NF-κΒ and IL-6 was detected.

**Conclusions:**

NF-κΒ is activated in the early stages of oral carcinogenesis. IL-6 may have an NF-κΒ-independent role, possibly through regulation of the inflammatory response.

**Key words:**NF-κB, IL-6, immunohistochemistry, oral squamous cell carcinoma, oral precancerous lesion.

## Introduction

The study of potentially malignant disorders offers insight into the pathogenesis of oral cancer. Furthermore, continuing investigation of the molecular basis of oral carcinogenesis may help to distinguish lesions with increased malignant potential and to discover new targets for molecular chemotherapy. It is widely known that carcinogenesis may occur through evolution of pre cancerous lesions or conditions (1). A pre cancerous lesion is a morphologically altered tissue in which oral cancer is more likely to occur than in its apparently normal counterpart (2). The most common pre cancerous lesions in oral mucosa are leukoplakias and erythroplakias (1).

The role of inflammation is implicated in the pathogenesis of cancer. It is estimated that 15-20% of all cancers are etiologically associated with inflammation, especially of chronic type (3). Tumor cells are known to produce inflammatory agents in order to alter the stroma and facilitate invasion (4). Increased inflammatory cytokine levels are found in many cancer cell lines including oral squamous cell carcinoma (OSCC) (5). Laboratory trials showed that even acute inflammation may lead to increased invasion and metastases (3). Inflammation is also related to the expression of several oncogenes, such as RAS and MYC (6).

The transcription factor Nuclear Factor-kappa B (NF-κB) has been extensively studied in carcinogenesis (7). Mammalian NF-κB protein family is composed of five members, RelA (p65), RelB, cRel (Rel), NF-κB1 (p50 and its precursor p105) and NF-κB2 (p52 and its precursor p100) (8). These molecules form homodimeric and heterodimeric complexes, the activity of which is regulated by different pathways (8). The classical pathway of NF-κB is the most commonly studied due to its major role in the control of innate immunity and inflammation (8). It is modulated by the p65:p50 dimer, which is constantly inactive in the cytoplasm bound to inhibitory molecules (Inhibitors of κΒ–ΙκΒs) (8). Various conditions initiate the degradation of the IκΒs, so that the ΝF-κΒ complex is freed to enter the nucleus and activate target genes (8). Specifically, NF-κΒ regulates the expression of proteins that have prominent roles in cell proliferation, survival, immune response and inflammation, such as Interleukin-6 (IL-6) (7,9).

IL-6 is a pro-inflammatory cytokine with autocrine and paracrine functions (10). It binds to a membrane receptor, which is a heterodimer-composed of IL-6 special receptor protein (IL-6Ra/gp80 or CD126) and gp130 (CD130) (10). IL-6 receptor (IL-6R) activates the Janus kinase family (JAK1, JAK2, TYK2) via gp130 (10). In turn, these kinases activate the molecular pathways of Signal transducer and activator of transcription 3 (STAT3), Phosphoinositide 3-kinase (PI3K) and Mitogen-activated protein kinases (MAPKs) with various oncogenic consequences (4,10).

Currently, no studies have explored the immunohistochemical correlation between NF-κB and IL-6 in oral pre cancerous lesions. The aim of this study was to evaluate the immunohistochemical expression of NF-κB and IL-6 in oral pre cancerous lesions (leukoplakias and erythroleukoplakias) and to investigate their possible correlation with the presence of subepithelial inflammatory infiltrate.

## Material and Methods

* Material

Forty five biopsies were retrieved from the files of the Department of Oral Medicine and Pathology, Dental School, National and Kapodistrian University of Athens. Thirty two oral pre malignant lesions, clinically compatible with leukoplakia or erythroplakia, were investigated. Microscopically, 11 of them showed hyperkeratosis and acanthosis (epithelial hyperplasia) and 21 showed dysplasia of varying degrees. Nine cases of OSCC and four control cases of normal oral mucosa were also included in the study. The histological features were reviewed for confirmation of the diagnosis according to the WΗΟ 2005 guidelines. The research protocol was IRB approved.

The available epidemiological and clinical characteristics were collected. All precancerous lesions presented clinically as leukoplakia or erythroleukoplakia without a history of previous interventions. The normal mucosa cases were harvested from sites adjacent to reactive oral mucosal lesions in patients with no medical history of oral pre malignancy or malignancy and no social history of smoking or excessive alcohol drinking.

* Methods

- Immunohistochemistry 

Paraffin-embedded tissue sections of 5μm thickness were deparaffinized (5 min in 60oC), immersed in xylene and ethanol solutions, and heated for antigen retrieval in 0.01 M citrate buffer for 15 min in a pressure cooker inside a microwave oven. After incubation in 3% hydrogen peroxide (LSAB+ kit Dako Corporation, Carpinteria USA) to neutralize endogenous peroxidase activity and treatment with Protein Free Serum (LSAB+ kit Dako Corporation, Carpinteria USA) to reduce nonspecific binding of primary antibody and polymer, the sections were incubated with primary antibodies overnight. The applied antibodies were monoclonal anti-p65 mouse antibody (sc-8008, Santa Cruz Biotechnology, Santa Cruz, California, USA) diluted at 1:200 and monoclonal anti-IL-6 mouse antibody (clone 6708.11, Sigma, Saint Louis, Missouri, USA) diluted at 1:25. Standard streptavidin-biotin-peroxidase complex method was employed to bind to the primary antibody along with multilink concentrated biotinylated anti-IgG as secondary antibody (LSAB+ kit Dako). Reaction products were visualized by staining with 3,3V-diaminobenzidine reagent (LSAB+ kit Dako). Sections were counterstained with hematoxylin. As positive controls, the following were used: OSCC for p65 and papillary thyroid carcinoma for IL-6 with corresponding known positivity. As a negative control, sections were treated with PBS, with the omission of the primary antibody.

- Immunohistochemical scoring 

**Figure 1:**
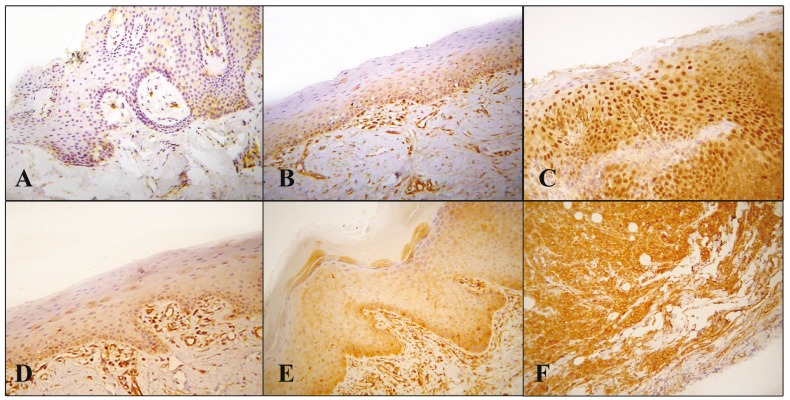
Cytoplasmic NF-κΒ (p65) staining grades according to the percentage of positive epithelial cells [1, < 20% (A); 2, 20-50% (B); 3, > 50% (C)] and the intensity of staining [1, weak (D); 2, moderate (E); 3, strong (F)]. Note the prominent positive nuclear staining in C. (A-F: 100X).

Immunostains were reviewed by three independent evaluators (N.N., G.K. and A.S.). Immunohistochemical reactivity for NF-κB (p65) was assessed separately in the cytoplasm and in the nucleus of the epithelial cells since it has been suggested that this molecule’s localization may represent a transition between inactive and active forms (8). NF-κB cytoplasmic staining was graded according to the percentage of positive epithelial cells (0, 0%; 1, < 20%; 2, 20–50%; 3, > 50%) (Fig 1A-C) and intensity of staining (0, no staining; 1, weak; 2, moderate; 3, strong) (Fig 1D-F) compared to negative control tissues; a combined score (0-6) was also calculated. NF-κB nuclear staining in the epithelial cells was characterized as positive or negative (due to primarily focal localization, no further assessment was performed). Immunohistochemical cytoplasmic reactivity for IL-6 was characterized as positive or negative.

- Assessment of inflammation

Inflammation was microscopically assessed on hematoxylin and eosin stains. If sub epithelial inflammatory infiltrate (mild to severe) was noted, the case was characterized as positive. The absence of sub epithelial inflammation resulted in the characterization of the specific case as negative.

- Statistical analysis

The baseline characteristics of patients were summarized as mean and standard deviation (SD) for continuous or ordinal data and as absolute (n) and relative (%) frequency for categorical variables. The two tailed Fisher’s exact test was performed in order to evaluate possible differences in the frequency distribution of clinical and pathologic features of patients or the parameters of p65 immunohistochemical expression, according to the histological classification of lesions.

The same test was also performed to evaluate possible associations between various parameters of IL-6 immunohistochemical expression and the presence of inflammation. The Mantel-Hæszel method was then applied for the calculation of odds ratios (OR) and their respective 95% confidence intervals (95% CI) in 2 x 2 contingency tables, wherever possible. Pearson Correlation Coefficients (r) were calculated between all dichotomous variables and inflammation presence, as well.

Comparisons concerning the age of patients were based on one-way ANOVA, while ordinal data were compared with the use of the Kruskal-Wallis one way analysis of variance by ranks. Post - hoc pairwise comparisons after a significant Kruskal-Wallis test were carried out with the application of Dunn’s test.

Statistical analyses were performed using the SPSS software application (version 21.0: SPSS, Chicago, IL, U.S.A.) with *p*<0.05 as the threshold of significance.

## Results

- Cohort characteristics

Distribution of the demographic characteristics (sex, age and location) according to the histologic type is presented in  Table 1. No statistically significant difference was observed in the distribution of these parameters between different histologic types.

**Table 1:**
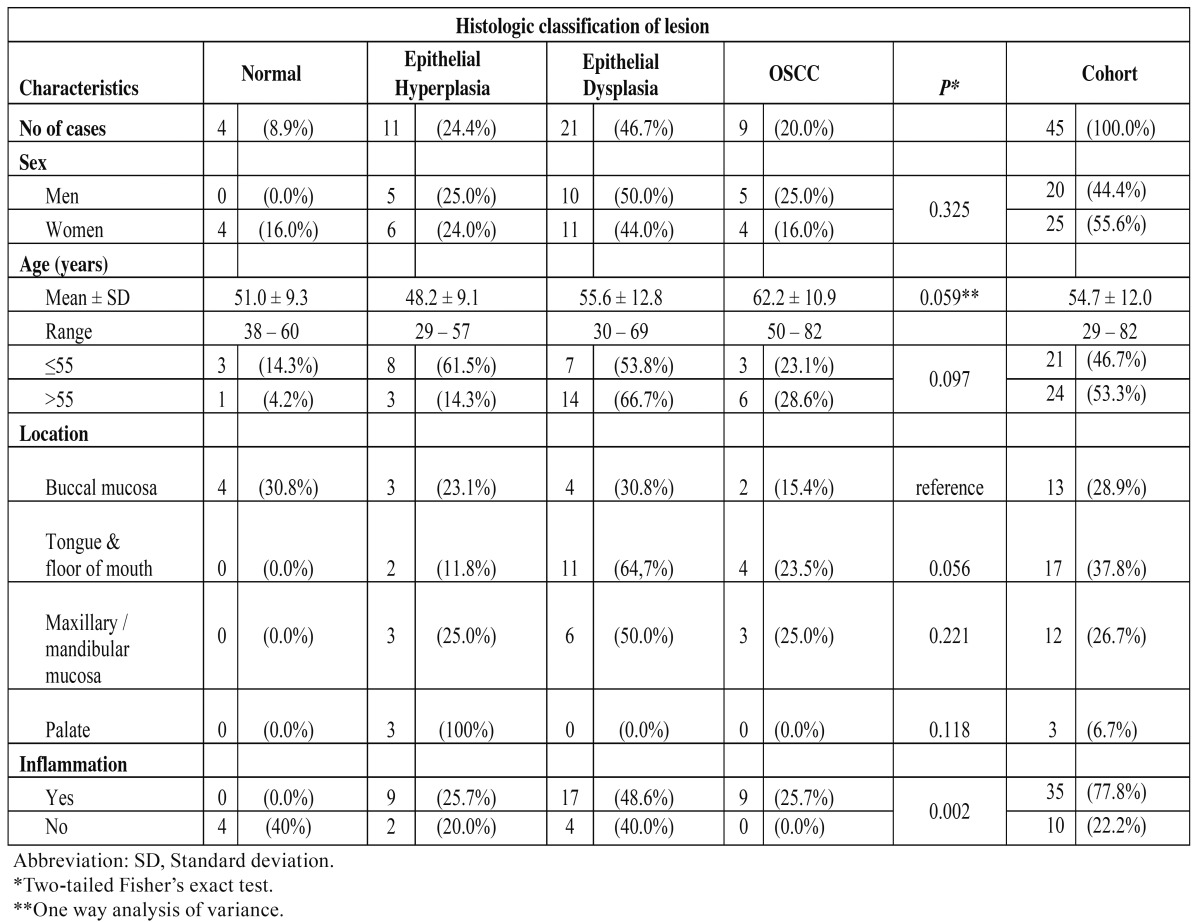
Patients’ clinical characteristics and presence of inflammation per histologic type of lesions.

- Presence of inflammation

Sub epithelial inflammatory infiltrate (primarily of chronic nature) was observed in 0/4 cases of normal epithelium (0%), 9/11 cases of epithelial hyperplasia (81.8%), 17/21 cases of dysplasia (81%) and 9/9 cases of OSCC (100%) (*p*=0.002) ( Table 1). Compared with normal mucosa, the presence of inflammation was higher in epithelial hyperplasias (*p*=0.011, 95% confidence interval [CI] lower border 2.95), dysplasias (*p*=0.006, 95% CI lower border 3.41) and OSCCs (*p*=0.001, 95% CI lower border 9.93).

- NF-κΒ immunohistochemical expression

**Figure 2:**
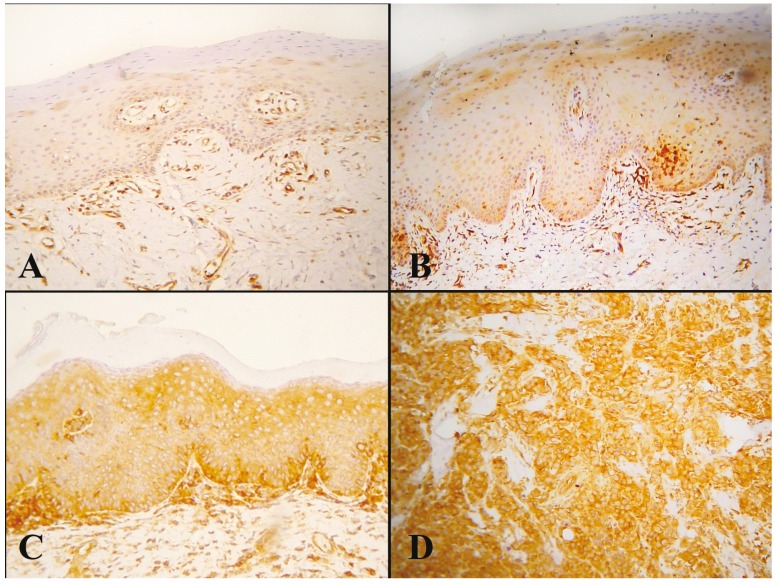
NF-κΒ (p65) immunohistochemical staining in normal tissue (A), epithelial hyperplasia (B), epithelial dysplasia (C) and OSCC (D), showing a gradual increase in cytoplasmic intensity from normal to cancer. (A-D: 100X).

NF-κB (p65) cytoplasmic expression was detected in the epithelial cells of all cases; as expected, p65 expression was also seen in the subjacent inflammatory cells. The average percentage, intensity and total scores for NF-κB cytoplasmic expression in the epithelial cells are presented in  Table 2. No statistical significant difference was detected in the percentage score for p65 positive cells. On the other hand, average intensity scores showed a statistically significant gradual increase from normal mucosa to OSCC (*p*=0.012) ( Table 2) (Fig. 2). Post-hoc analysis revealed that the greatest difference was observed in the transition from epithelial hyperplasia to dysplasia (*p*=0.011). Moreover, total scores for NF-κB cytoplasmic expression also showed a similar (to intensity) statistically significant difference between the histological categories (*p*=0.026) ( Table 2).

**Table 2:**
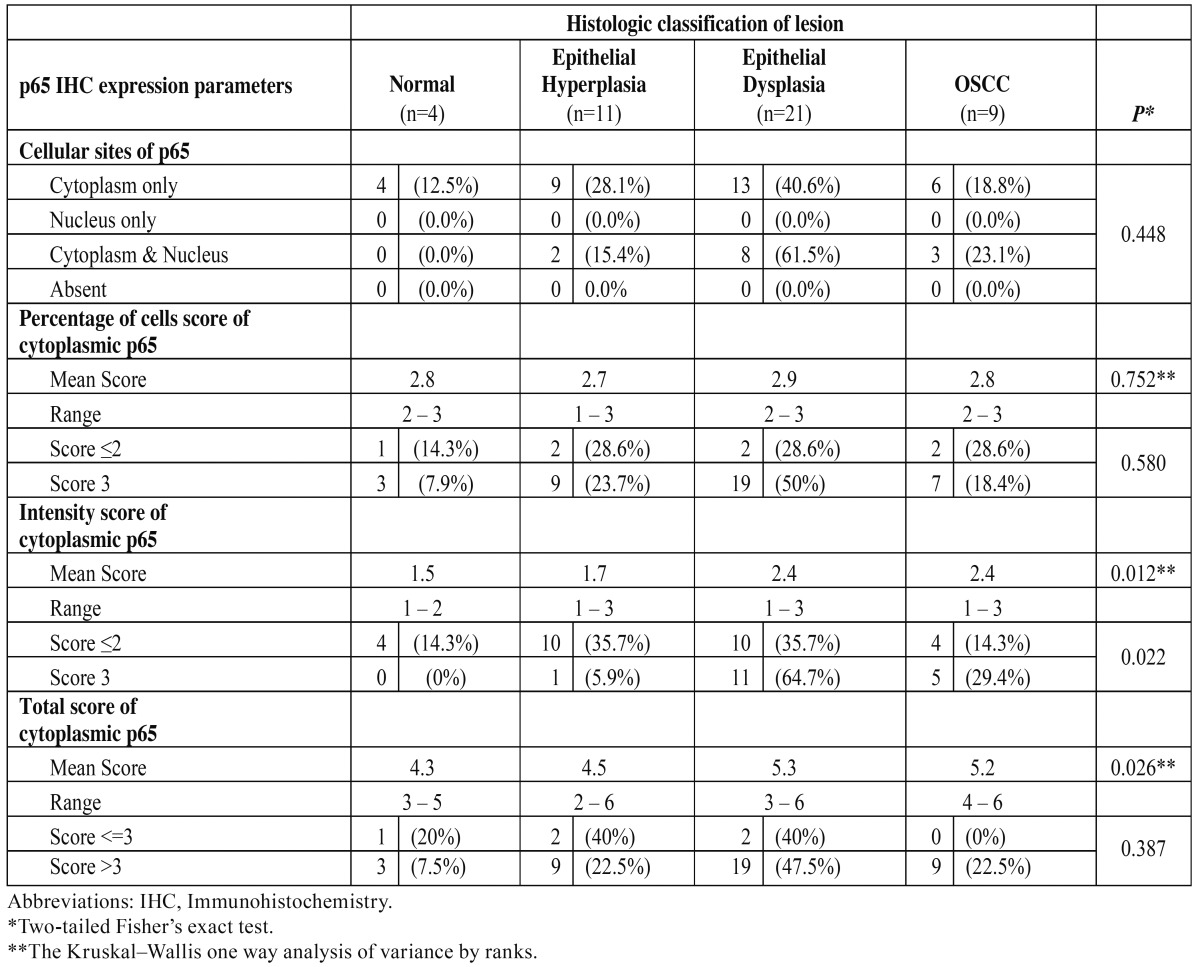
Comparison of p65 immunohistochemical parameters between different histologic types of lesions.

 NF-κΒ nuclear localization was detected in clusters of cells in 0/4 (0%) of the normal mucosa cases, 2/11 (18.2%) of the epithelial hyperplasia cases, 8/21 (38.1%) of the dysplasia cases and 3/9 (33.3%) of the OSCC cases ( Table 2) (Fig. 1C). Even though there seems to be an increased number of cases exhibiting nuclear localization among dysplasias and OSCCs, no statistical significant difference was observed.

There was no statistical correlation between the expression of NF-κΒ (p65) and the presence of the sub epithelial inflammatory infiltrate (data not shown).

- IL-6 immunohistochemical expression 

**Figure 3:**
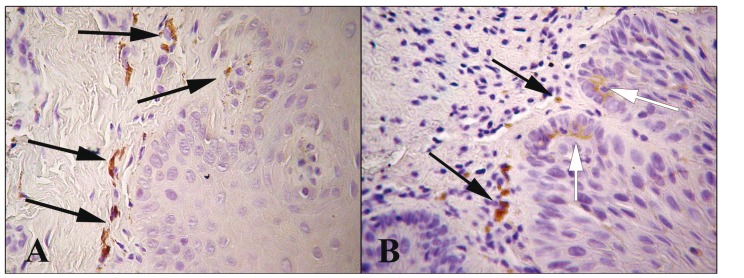
IL-6 immunohistochemical staining in subepithelial inflammatory cells (black arrows) and in the adjacent epithelial cells of the basal or parabasal layer (white arrows) of selective cases. (A-B: 400X).

IL-6 had a generally mild cytoplasmic staining in a few subepithelial inflammatory cells and in the adjacent epithelial cells of the basal or parabasal layer of selective cases (Fig. 3).

Specifically, IL-6 expression was detected in 3/11 (27.3%) of the epithelial hyperplasia cases, 10/18 (55.6%) of the dysplasia cases and 2/7 (28.6%) of the OSCC cases, whereas no positive case was detected in normal oral mucosa. No statistical difference was observed between the above groups. Overall, epithelial dysplasias showed non-statistically significant increased positivity for IL-6 compared to hyperplasias and OSCCs.

There was no statistical correlation between the expression of IL-6 and the nuclear or cytoplasmic expression of NF-κΒ (p65). On the other hand, positive statistical correlation between the presence of inflammation and the expression of IL-6 was detected (*p*=0.015, 95% confidence interval [CI] lower border 1.99) (Table  Table 3).

**Table 3:**
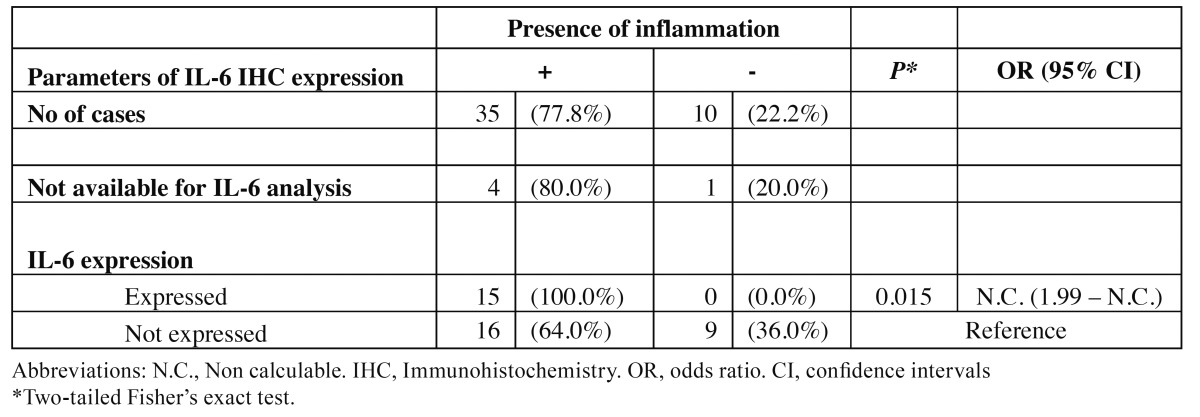
Correlation of IL-6 immunohistochemical expression with inflammation.

## Discussion

The role of chronic inflammation in oral carcinogenesis is not fully understood. Transcription factor NF-κΒ is known to have prominent role in the inflammatory response (3,9). Moreover, pro inflammatory cytokines, such as IL-6, are often involved in the interaction between epithelial and stromal cells (6,9). The current study’s objective was to evaluate the expression of these key molecules in oral pre cancerous and cancerous lesions and correlate it with each other, with the histologic subtype of the lesions and with the presence of sub epithelial inflammatory infiltrate.

NF-κΒ pathway is implicated in oral carcinogenesis. Among its family members, p65 is the most frequently studied. It is part of the p65(RelA):p50 dimer which mediates the classical pathway. In the present study, p65 was expressed in the cytoplasm of the epithelial cells in every case. This finding may be attributed to the sensitivity of the anti-p65 antibody used. The same antibody was used in other studies also, revealing high p65 expression rates (7,11). It should be noted that the cytoplasmic expression of p65 in OSCC is over 75% in various studies, while nuclear staining is detected in just 35-45% of the cases (7,11-16). In the present study, nuclear localization was detected in 33.3% of OSCC cases. As for the oral pre cancerous lesions, it is generally believed that p65 expression (percentage and intensity) gradually increases from normal mucosa to epithelial dysplasia (13-16). In the present study, p65 cytoplasmic intensity and total scores showed a statistically significant gradual increase from normal mucosa to OSCC, while non-statistically significant increased nuclear localization was detected in epithelial dysplasias and OSCCs. These findings suggest that p65 may be expressed in all stages of oral carcinogenesis with increased expression especially in the transition between oral epithelial hyperplasia and dysplasia.

Chronic inflammation and IL-6 in particular, are involved in colon and prostate carcinogenesis (17-19). In the present study, the non-statistically significant increased IL-6 expression in epithelial dysplasias compared to hyperplasias and normal mucosa suggests a possible role of this molecular pathway in oral carcinogenesis. Interestingly, no other studies have tested IL-6 immunohistochemical expression in oral pre cancerous lesions. As for OSCC, only 2/7 cases (28.6%) were positive for IL-6. Other immunohistochemical studies have found that 35.7-100% of OSCC tissues or cell lines express IL-6 (20-24). This disagreement can be attributed to the small sample (n=7) of the research. On the other hand, these tumors may still express IL-6R in order to receive IL-6 signaling from the subepithelial inflammatory infiltrate, as suggested by other studies (24).

In the present study, increased presence of inflammation was detected in epithelial hyperplasias, dysplasias and OSCCs, compared with normal mucosa. In addition, a statistically significant correlation between IL-6 expression and the presence of sub epithelial inflammatory infiltrate was observed. These findings should be interpreted in the context of a possible crosstalk between the epithelium and the stroma in oral carcinogenesis. The inflammatory cells (macrophages and lymphocytes) typically produce IL-6 that binds to the epithelial cells via IL-6R and/or sIL-6R, thus facilitating cellular growth and invasion (4,9,25,26). Moreover, inflammatory cells also express IL-6R and are able to receive signals from the epithelium (27,28). These molecular signals cause oncogenic differentiation of the inflammatory infiltrate towards Th2 lymphocytes and M2 macrophages (29). The exact role of IL-6 in the early stages of oral carcinogenesis, before invasive carcinoma is formed, needs further investigation.

The expression of IL-6 is commonly regulated by NF-κB (7). In the present study, no correlation between the expression of IL-6 and the nuclear or cytoplasmic expression of NF-κΒ (p65) was noted. This fact indicates that NF-κΒ may act in an IL-6-independent fashion by activating different targets in oral carcinogenesis, such as the anti-apoptotic protein Bcl-2 and various other cytokines and chemokines (30). Moreover, IL-6 might be activated by molecules other than NF-κΒ, such as Interleukin-1 (IL-1), Tumor necrosis factor (TNF), prostaglandin E-2 (PGE-2) and vascular endothelial growth factor (VEGF) (9).

The results of this study support that the signaling pathway of NF-κΒ is activated in the early stages of oral carcino-genesis. At the same time, IL-6 might have an independent role in the regulation of the inflammatory response. Interestingly, the intensity of NF-κΒ expression is significantly increased in the transition from epithelial hyperplasias to dysplasias. The increased presence of inflammation in epithelial hyperplasias, dysplasias and OSCCs and its statistical correlation with IL-6 expression is consistent with a possible crosstalk between the epithelium and the stroma. On the other hand, the absence of correlation between NF-κB expression and IL-6 expression suggests that this cytokine may be the downstream target of other NF-κB-unrelated molecular pathways. Further studies regarding these processes may be of significance in the quest for better prognostication and/or management of potentially malignant disorders of the head and neck.
